# Prospective Trial of a Novel Nomogram to Achieve Updated Vancomycin Trough Concentrations

**DOI:** 10.1155/2013/839456

**Published:** 2013-09-12

**Authors:** Amber R. Wesner, Marcia L. Brackbill, Larissa L. Coyle, Robert S. Kidd

**Affiliations:** ^1^Department of Pharmacy Practice, Shenandoah University, 1775 North Sector Court, Winchester, VA 22601, USA; ^2^Department of Pharmacy, Winchester Medical Center, 1840 Amherst Street, Winchester, VA 22601, USA; ^3^Department of Biopharmaceutical Sciences, Shenandoah University, 1775 North Sector Court, Winchester, VA 22601, USA

## Abstract

*Purpose*. To determine if the use of a novel vancomycin nomogram predicts dosing regimens that achieve target trough concentrations equal to or more accurate than dosing regimens calculated using traditional pharmacokinetic calculations, evaluate the incidence of subtherapeutic and supratherapeutic troughs, and assess pharmacist's impressions of the nomogram. *Methods*. Prospective, open-label study in 473 patients who had a new order for vancomycin and were >18 years of age and ≤120 kg. Patients were randomized to the active group, dosed using the nomogram, or to the control group, dosed using traditional pharmacokinetic calculations already in place at our institution. *Results*. Patients dosed via nomogram were within the appropriate trough range in 44% of cases compared to 33% in the control group (*P* = 0.014). Vancomycin troughs less than 10 mcg/mL were significantly decreased with the use of nomogram (*P* = 0.032). Incidence of supratherapeutic troughs, greater than 20 mcg/mL, was not significantly different between groups (*P* = 0.706), and pharmacists agreed that the nomogram was easy to use and saved their time. *Conclusions*. A novel vancomycin nomogram was prospectively validated and found to be more effective than traditional pharmacokinetic dosing. The nomogram is being implemented as the standard dosing protocol at our institution.

## 1. Introduction

Vancomycin is a glycopeptide antibiotic used to treat infections caused by gram-positive organisms, most commonly methicillin-resistant *Staphylococcus aureus* (MRSA). It is used for a variety of infections including cellulitis, pneumonia, sepsis, and endocarditis [1]. Due to potential side effects such as nephrotoxicity, ototoxicity, and the potential for bacterial resistance with low troughs, vancomycin concentrations are routinely monitored to ensure appropriate dosing. Previously, appropriate trough concentrations were considered to be between 5–15 mcg/mL [2, 3]. However, due to the emergence of vancomycin resistance, recommended trough concentrations have increased.

In 2009, the American Society of Health-System Pharmacists/Infectious Diseases Society of America/Society of Infectious Diseases Pharmacists published a consensus statement, updating the guidelines for appropriate vancomycin trough concentrations [2]. These guidelines recommend that troughs should remain above 10 mcg/mL at all times to limit treatment failures and resistance. For patients being treated for severe infections such as, bacteremia, endocarditis, meningitis, MRSA pneumonia, osteomyelitis, or sepsis, trough concentrations should be maintained between 15 and 20 mcg/mL [2]. These elevated trough concentrations are needed due to the emergence of vancomycin-resistant *Staphylococcus aureus *classified as vancomycin-intermediate *Staphylococcus aureus *(VISA) or vancomycin-resistant *Staphylococcus aureus * (VRSA), based on reported minimum inhibitory concentrations (MICs). There is also a heteroresistant *Staph aureus* (hVISA) that shows inducible resistance after exposure to vancomycin [2, 4]. The purpose of elevated troughs is to prevent further emergence of resistance and provide better treatment of infection.

In order to achieve these updated trough goals, traditional vancomycin dosing may not be adequate. Existing nomograms such as the Matzke, Lake and Peterson, Rotschafer, Nielson, and Moellering were not designed to achieve troughs greater than 15 mcg/mL, and most were actually designed to achieve troughs of 10 mcg/mL or less [5–9]. The use of these nomograms would result in subtherapeutic vancomycin levels, which makes these historical nomograms obsolete. Therefore, there is a need to develop new nomograms, designed to achieve the updated trough guidelines.

Currently, several dosing strategies that have been published aimed at achieving these higher trough levels, each with varying limitations [10–12]. The nomogram by Kullar et al. was designed to achieve troughs between 15 and 20 mcg/mL and was found to be effective in 58% of patients in which it was studied [10]. This study excluded patients if they weighed >110 kg, had a creatinine clearance <30 mL/min or >110 mL/min, or were considered to be critically ill, limiting the broad application of this nomogram. The second dosing protocol by Devabhakthumi et al. was a retrospective analysis utilizing the patient's weight to determine dose and renal function to determine the dosing interval. Although results showed that an increased number of patients were initiated on an appropriate vancomycin dose, which authors defined as 15 mg/kg/dose, the percentage of patients achieving appropriate trough concentrations was not improved with the implementation of the dosing protocol [11]. Also, no adjustment was made to the dosing protocol if the patient was obese or nonobese. The last, the nomogram by Thalakada et al. utilized multiple regression analysis in order to provide equations for calculating a dosing interval, using age and serum creatinine as variables, which aimed at achieving a vancomycin trough of 15–20 mcg/mL [12]. It was found to be effective in 56% of patients. This nomogram only addresses the vancomycin dosing interval that is required, and it can only be used to achieve trough concentrations between 15 and 20 mcg/mL. Also, a majority of patients included in the development of the nomogram (86%) had initial serum creatinine measurements between 0.5 and 1.6 mg/L, limiting the range of renal function to which the nomogram can be generalized [12].

Two retrospective investigations were previously completed at our institution to evaluate a novel vancomycin nomogram, designed to achieve the higher vancomycin trough goals. One investigation was completed for nonobese patients, and the second evaluated an obese population. Obesity was defined as a weight 30% or more over ideal body weight (IBW). Based on results of these studies, the new vancomycin nomogram (see (1) and Tables 4 and 5) has been shown to have a high level of accuracy in predicting doses of vancomycin that are appropriately therapeutic and have been validated retrospectively.

*Vancomycin Nomogram.* Consider the following

(1) IBWmale=50+2.3(inches over 5′ tall),IBWfemale=45.5+2.3(inches over 5′ tall),CrCL=(140−age)(IBW or DW if obese)(72)(Scr)(× 0.85 for females),Percent over IBW=ABW−IBWIBW×100%,DW=IBW+0.4(ABW−IBW).

The loading and maintenance doses are calculated as shown in Table 4.

The dosing intervals are based on the estimated CrCl and are determined as shown in Table 5.

The purpose of this study was to prospectively evaluate the novel vancomycin nomogram in both obese and nonobese patients. By further evaluation of this promising nomogram, we aimed to provide a tool for clinicians which may result in easier and more accurate dosing to achieve the updated trough goals and to reduce the time spent on pharmacokinetic dosing of vancomycin.

The primary objective of this study was to determine if the novel vancomycin nomogram predicts dosing regimens that achieve target trough concentrations equal to or more accurate than dosing regimens calculated using traditional pharmacokinetic calculations by evaluating the percentage of patients achieving initial trough concentrations within the goal range based on the patient's indication for therapy.

Secondary objectives of the study were to assess the incidence of subtherapeutic (<10 mcg/mL) and supratherapeutic (>20 mcg/mL) trough measurements and to assess pharmacist's impressions of the nomogram.

## 2. Materials/Methods

This prospective, open-label trial was performed at a 454-bed community hospital on patients who were newly started on vancomycin therapy. Patients were included if they were greater than 18 years of age, less than or equal to 120 kg, and had a new order for vancomycin where pharmacy was consulted to dose and monitor therapy. Exclusion criteria included age less than 18 years, pregnancy, end stage renal disease requiring dialysis or any renal replacement therapy, weight greater than 120 kg, an increase in serum creatinine of 50% or more from baseline during the course of therapy, and patients with anasarca. This study was approved and was compliant with the ethical standards of Winchester Medical Center's Institutional Review Board and adhered to the pharmacy departments approved vancomycin dosing policy.

Pharmacists who received a vancomycin consult would determine a patient's eligibility for study inclusion and randomize the patient, using a vancomycin randomization form provided. Randomization sheets were created using a random number generator. The treatment group was dosed using the novel nomogram and the control group was dosed using the traditional pharmacokinetic methods currently used for vancomycin dosing at the institution. Traditional pharmacokinetics was defined as calculating a vancomycin dose in whichever manner the pharmacist utilized prior to the start of the study. This included using the dosing equations provided by the pharmacy department or a historical nomogram already used by the pharmacists (see supplementary material Appendix 1 available online at http://dx.doi.org/10.1155/2013/839456). All pharmacists were educated on how to screen patients for inclusion, how to use the randomization form, and how to use the nomogram to include necessary monitoring and followup. All pharmacists were educated by a single investigator to ensure that the same content was relayed to each pharmacist. Inservices and an instructional notebook were provided to limit interclinician variability. After randomization, a standard data collection and monitoring form was filled out for each patient. Separate forms were used based on the patient's study arm (see supplementary material Appendix 1). Each form was organized in a step-by-step scripted format to ensure the consistent use of the nomogram and pharmacokinetic calculations between pharmacists.

In order to assess pharmacist's impressions of the nomogram, surveys were distributed to the entire pharmacy department. Pharmacists were asked if they felt that the nomogram was easy to use, saved them time, could improve patient care, if they felt comfortable using the nomogram, and if they were satisfied with the nomogram. The survey was optional and anonymous for pharmacists to complete. The baseline survey was administered during the first week of the study, and the final survey was administered at the conclusion of data collection.

Patients were enrolled from January 10, 2011 to October 31, 2011. For patients to be included in the final analysis, they must have received three identical vancomycin doses and have a trough level drawn at steady state (just prior to the fourth or subsequent doses), and the nomogram must have been used correctly if used in the active nomogram group. Correct use of the nomogram was defined as accurately calculating the patient's vancomycin dose and correctly selecting the dosing interval based on guidelines provided with the nomogram. Doses were capped at 2,000 mg per dose based on our institutions standard practice. A trough could have been drawn prior to steady state, if deemed clinically indicated by the pharmacist, but any changes to the dosing regimen based on this trough measurement will result in the exclusion of that patient from data analysis. Extrapolated vancomycin troughs were calculated for all measured troughs to be used in final analysis. An extrapolated trough was defined as the vancomycin trough measurement just before the next dose of vancomycin was due. In determining if a trough measurement was within the appropriate range, extrapolated troughs were calculated out to one decimal place, not rounded, and had to fall within the absolute limits of 10.0–15.0 or 15.0–20.0 mcg/mL, depending on the trough goal classification. To detect a 20% difference between the nomogram and traditional pharmacokinetic dosing methods, with an 80% power, 414 patients were needed in the final analysis.

Data were analyzed using SPSS (SPSS statistics, version 19). Categorical data, the primary objective, occurrence of patients achieving target trough measurements, and the secondary objective, the occurrence of subtherapeutic and supratherapeutic troughs, were evaluated using the Chi-square test. Survey data, evaluating pharmacist's opinions of the nomogram on a 5-point likert scale, were analyzed using the Wilcoxon test. A *P* value less than 0.05 was considered significant.

## 3. Results and Discussion

### 3.1. Results

**Figure 1:**
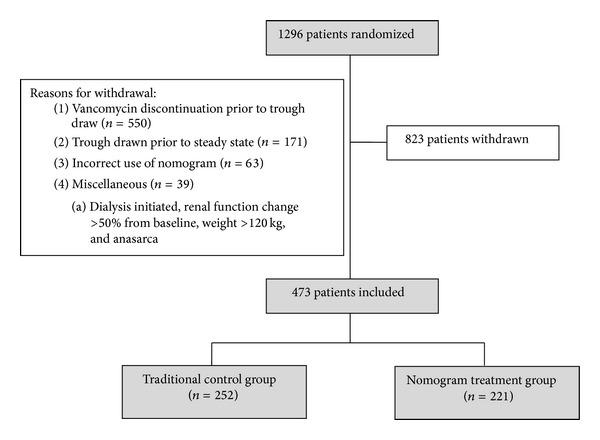
Study arms.

One thousand and two hundred ninety-six patients were randomized into the study, of which, 823 patients had to be withdrawn from data analysis. Reasons for withdrawal included vancomycin therapy being discontinued prior to an initial trough draw, troughs drawn prior to steady state, and incorrect use of the nomogram (Figure 1). Four hundred seventy-three patients were included in the final analysis.

Demographic data are presented in Table 1. Patients were similar at baseline with the exception of age. The average age in the nomogram group was 58 ± 18 years versus 61 ± 17 years in the traditional group (*P* = 0.040). Baseline renal function and change in renal function during vancomycin therapy were similar between groups. Additionally, approximately 20% of each group included patients who were critically ill.

**Figure 2:**
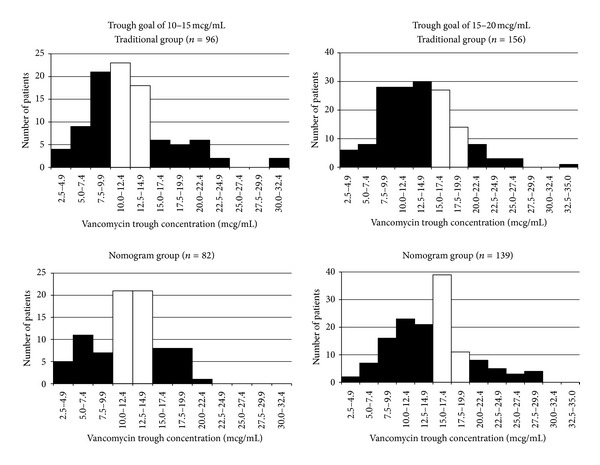
Distribution of vancomycin trough concentrations among treatment groups and target trough ranges. White bars represent patients in target trough range, and black bars represent patients who were out of the target range.

Overall, the nomogram resulted in significantly more patients achieving an appropriate vancomycin trough concentration with their initial dosing regimen (*P* = 0.014), which was the primary endpoint of the study. In the nomogram group, 98 out of 221 patients (44%) achieved a therapeutic vancomycin trough compared to 83 out of 252 patients (33%) in the control group. The distribution of all trough measurements is displayed in Figure 2. 

A subgroup analysis, evaluating only patients dosed via the nomogram, was performed. Patients were stratified based on if they were classified as obese or nonobese. In obese patients, the nomogram achieved a therapeutic vancomycin trough in 43 of 109 patients (39%) compared to nonobese patients where the nomogram achieved therapeutic troughs in 55 out of 112 patients (49%). Although the nomogram appears to have a higher level of accuracy among nonobese patients, the difference was not statistically significant (*P* = 0.190), and the study was not powered to detect a difference between these groups.

Data for vancomycin concentrations below 10 mcg/mL and above 20 mcg/mL are presented in Table 2. The nomogram group had significantly fewer trough concentrations less than 10 mcg/mL compared to the traditional group (20.8% versus 29.9%, resp., *P* = 0.032). However, there was no significant difference between the groups for concentrations above 20 mcg/mL (8.1% in the nomogram group versus 9.6% in the traditional group, *P* = 0.706).

Survey data representing pharmacist's impressions of the nomogram are presented in Table 3. From baseline to the conclusion of the study, significant improvements were seen in the ease of use (*P* = 0.033) and time saving (*P* = 0.008) aspects of the nomogram. Also, on the final survey, an additional question was added asking pharmacists if they would prefer the nomogram to be available for future use within the department. Twelve of the fourteen pharmacists responded that yes they would like the nomogram to be available, while the remaining two did not answer the additional question.

## 4. Discussion

Due to updates in the recommended vancomycin trough concentrations and the lack of validated nomograms designed to achieve these new recommendations, research is currently focused on developing new nomograms to assist clinicians in dosing vancomycin. Previously, at our institution, two vancomycin nomograms have been retrospectively validated. One nomogram for nonobese patients, and one nomogram was specifically modified for obese patients. The present study was designed to prospectively evaluate the combined nomogram and found the nomogram to be significantly more accurate when compared to traditional dosing completed by pharmacists at our institution. Nomogram dosing was able to achieve therapeutic vancomycin troughs in a greater percentage of patients compared to traditionally dosed patients.

The nomogram was also able to significantly reduce the incidence of subtherapeutic (<10 mcg/mL) vancomycin trough measurements. This trend for under dosing in the traditional group, which was especially prevalent in the 15–20 mcg/mL group was decreased when the nomogram was used as depicted in Figure 2. Since subtherapeutic vancomycin troughs are associated with treatment failure and the development of resistance, this finding supports the implementation and use of the nomogram to help reduce the incidence of these unwanted, serious, and costly outcomes. 

In demographic analysis, we found a statistically significant difference in age between treatment groups. Patients were randomly assigned to study groups so the reason for this difference is unknown. However, the average three-year age difference between groups is not considered clinically significant by the authors and does not affect the interpretation of the results overall.

The acceptance and ease of use of the nomogram were also evaluated by surveying pharmacist's impressions. At baseline, pharmacists were neutral or agreed with all statements asked. At the conclusion of the study, pharmacists fully or strongly agreed with all statements. The most significant change seen was with regard to saving the pharmacists' time. At baseline, pharmacists were neutral on this point. However, after nine months of using the nomogram, they agreed that the nomogram did save them time when compared to traditional dosing. It is essential with any clinical tool that the end user feel confident when using it and that it enhances rather than hinders daily work and patient's care. Our findings, which show broad acceptance and support of the nomogram, indicate that the nomogram enhances the pharmacist's workflow in addition to being accurate.

The current study also found a trend of improved accuracy of the nomogram among nonobese patients compared to obese patients. This finding was not surprising due to the fact that it was consistent with findings from the retrospective validation of the nomogram. The nonobese nomogram had a higher correlation coefficient in the retrospective study in comparison to the obese nomogram, indicating that it would be more likely to predict doses with a higher level of accuracy.

The results of this study add to the current literature on the topic, which consists of few validated nomograms designed to achieve the new higher vancomycin troughs. In comparison to the nomogram developed by Kullar et al., which can be used to achieve troughs of between 15–20 mcg/mL, the present nomogram can be utilized to achieve troughs of 10–15 mcg/mL or 15–20 mcg/mL depending on the patients indication for vancomycin therapy [10]. Also, the nomogram in this study, investigated the populations excluded from the study conducted by Kullar et al. As stated in their limitations, the nomogram by Kullar et al. did not include patients >110 kg or with a creatinine clearance of <30 mL/min or >110 mL/min [10]. Our nomogram was studied in patients up to 120 kg and did not have an upper creatinine clearance cutoff for excluding patients. Only patients requiring renal replacement therapy or those with unstable renal function were excluded in the present study on the basis of renal function. In addition, Kullar et al. excluded critically ill patients, studying generally stable patients, a majority of whom were receiving vancomycin for empiric coverage [10]. Critically ill patients were included in the current study and constituted approximately 20% of each group. 

In comparison to the nomogram developed by Devabhakthumi et al., our nomogram is similar in design, utilizing weight to determine the vancomycin dose and creatinine clearance to determine the dosing interval [11]. However, the Devabhakthumi nomogram did not differentiate between obese and nonobese patients and also did not separate trough goals. A therapeutic trough in the Devabhakthumi et al. study was considered appropriate if it fell anywhere between 10–20 mcg/mL regardless of the patients' indication for therapy [11]. In contrast, our nomogram allows the clinician to choose the appropriate trough goal, 10–15 mcg/mL or 15–20 mcg/mL, based on the patient's indication for therapy. Our results are comparable to those in the Devabhakthumi et al. study, in which 44% versus 45% of subjects were in the therapeutic range, respectively, and this may be due to our similar inclusion criteria which was very broad with few exclusion criteria, making the nomograms broadly applicable [11]. Our results are lower than those seen in the Kullar et al. study, 44% versus 58%, respectively, and may be due to the fact that the patients included in the Kullar et al. study were generally stable. Kullar et al. excluded extremes of weight, renal function and critically ill patients [10]. Thus, it is difficult to generalize the findings of Kullar et al. to these populations. This may be the case for the Thalakada et al. nomogram as well. The nomogram developed by Thalakada et al. was designed to achieve troughs between 15 and 20 mcg/mL only, and a majority (86%) of patients included in the nomograms development had normal renal function, with serum creatinine measurements less than or equal to 1.6 mg/L [12]. It is also important to note that rounding was permitted within the Thalakada et al. study. Troughs between 14.5 and 20.5 mcg/mL were considered acceptable and therapeutic [12]. Rounding was not permitted in the present study. Absolute limits of 10.0–15.0 and 15.0–20.0 mcg/mL were used. Therefore, a trough measurement of 14.9 mg/dL was not considered to be at goal if the trough range was meant to be 15–20 mcg/mL, based on the patients indication. The use of rounding would have affected our results if we expanded our acceptable trough ranges. However, we chose to be strict in determining if a trough was therapeutic in order to provide a conservative estimate of the nomogram's accuracy and applied this method to both treatment groups. 

The current study has several limitations. First, it was an open-label study and pharmacists were aware of the group assignment for each patient. Pharmacists were also aware that the accuracy of their dosing would be evaluated through the study, which may have influenced the time and attention that pharmacists gave to traditional dosing responsibilities. We may have also experienced interclinician variability, particularly within our traditionally dosed group. Whether the more or less experienced pharmacists dosed a greater number of patients in our traditional group is unknown. If less experienced pharmacists dosed a greater number of patients, it is possible that the accuracy rate was lower than that if more experienced pharmacists completed a majority of the dosing consults. This potential error should have been minimized through the use of our standardized, step-by-step traditional dosing guidelines (see supplementary material Appendix 1). However, by providing this step-by-step dosing form, we may have also inadvertently improved the accuracy of our traditionally dosed group. The form was designed and provided to ensure that simplicity in incorporating the study into the pharmacist's daily work as well as to ensure all necessary information was provided for data collection. Nevertheless, by providing this scripted form, pharmacists were provided with all necessary equations needed to appropriately dose vancomycin for their patient. If we had not provided this form, accuracy within the traditionally dosed group may have been lower than what was observed.

The timing of trough draws and the administration times of vancomycin doses were also not always correct. At our institution, doses may be administered within one hour from the scheduled time, but doses administered outside this time cutoff are not uncommon. To control this factor, extrapolated troughs were calculated for all patients to ensure that patients were classified appropriately as to be in or out of goal.

It is also important to note that the current nomogram cannot be generalized to all populations. Based on our exclusion criteria, efficacy is unknown for patients weighing greater than 120 kg, pediatric patients, and those with rapidly changing renal function. Also, although we did not have a creatinine clearance cutoff for exclusion, we had few patients with creatinine clearance measurements of less than 40 mL/min. 

The nomogram has been validated, proven effective, and accepted as easy to use and time saving. It has been studied in a wide range of diagnoses, renal function, and weights, making it broadly applicable. Utilizing the nomogram for dosing could make completing pharmacokinetic consults more efficient thus freeing pharmacists for other clinical functions. Based on our current findings, the nomogram has been implemented as the standard dosing protocol for our institution. Future studies should be focused on further development of the obese nomogram due to our weight cap in this study and due to the fact that the obese nomogram did not perform as strongly as the nonobese nomogram in the subgroup analysis.

## 5. Conclusions

A novel vancomycin nomogram was prospectively validated and found to be more effective than traditional pharmacokinetic dosing. The nomogram, based off of weight and creatinine clearance, is adaptable and can be used in nonobese and obese patients, up to 120 kg. Also, the trough goal can be set at 10–15 or 15–20 mcg/mL based off of the patient's indication for therapy, making this a broadly applicable nomogram. Subtherapeutic trough measurements were significantly less common with nomogram dosing, and the nomogram was found to be easy to use and time saving by pharmacists. Implementation of the nomogram as the standard dosing protocol is warranted for our institution, but should not be used independently of clinical judgment.

## Supplementary Material

Appendix 1 contains examples of forms utilized during the study. Separate forms were provided for the nomogram and traditionally dosed groups. Also, separate forms were provided for obese and non-obese patients within the nomogram group.

## Figures and Tables

**Table 1:** Characteristics of study subjects.

Characteristics	Nomogram (*n* = 221)	Traditional (*n* = 252)	*P* value
Age (yrs)^a^	58 ± 18	61 ± 17	0.040
Actual body weight (kg)^a^	83.6 ± 18.7	83.9 ± 17.4	0.873
Ideal body weight (kg)^a^	63.5 ± 10.6	65.5 ± 11.6	0.051
Height (in)^a^	66.8 ± 3.9	67.5 ± 4.3	0.051
Initial SCr (mg/dL)^a^	0.96 ± 0.31	1.0 ± 0.38	0.143
Change in SCr (%)^a^	14 ± 11.6	15 ± 11.5	0.389
Intensive care unit admission: *n* (%)	40 (18)	57 (23)	0.271
Gender: *n* (% Females)	107 (48)	105 (42)	0.168
Patients stratified by creatinine clearance: *n* (%)			
≥100 mL/min	79 (35.7)	59 (23.4)	
80–99 mL/min	27 (12.2)	46 (18.3)	
60–79 mL/min	53 (24.0)	59 (23.4)	
40–59 mL/min	42 (19.0)	51 (20.2)	
20–39 mL/min	20 (9.0)	37 (14.7)	
≤19 mL/min	0	0	
Patients stratified by diagnosis: *n* (%)			
Pneumonia	66 (29.9)	76 (30.2)	
Cellulitis	68 (30.8)	72 (28.6)	
Bacteremia	32 (14.5)	32 (12.7)	
Osteomyelitis	11 (5.0)	13 (5.2)	
Abscess	15 (6.8)	11 (4.4)	
Empiric	10 (4.5)	16 (6.3)	
Urinary tract infection	2 (0.9)	9 (3.6)	
Respiratory failure	3 (1.4)	6 (2.4)	
Miscellaneous: endocarditis, meningitis, febrile neutropenia, diverticulitis, and wounds	14 (6.3)	17 (6.7)	

^a^Mean ± standard deviation.

SCr: serum creatinine.

**Table 2:** Occurrence of troughs less than 10 mcg/mL and above 20 mcg/mL.

Group	Nomogram (*n* = 221)	Traditional (*n* = 252)	*P* value
Troughs below 10 mcg/mL	46 (20.8%)	75 (29.9%)	0.032
Troughs above 20 mcg/mL	18 (8.1%)	24 (9.6%)	0.706

**Table 3:** Pharmacist survey evaluating opinions of nomogram.

Question^a^	Baseline mean ± SD (*n* = 13)	Conclusion mean ± SD (*n* = 14)	*P* value
The nomogram is easy to use	4.3 ± 0.48	4.8 ± 0.43	0.033
The nomogram has saved me time	3.5 ± 0.97	4.6 ± 0.76	0.008
The nomogram can improve patient care	4.1 ± 0.95	4.3 ± 0.83	0.616
I feel comfortable using the nomogram	4.2 ± 0.60	4.6 ± 0.50	0.116
I am satisfied with the nomogram	4.0 ± 0.89	4.6 ± 0.51	0.120

^a^Responses based on a 5-point likert scale (5: strongly agree and 1: strongly disagree).

**Table 4:** 

	Target trough 10–15 mcg/mL	Target trough 15–20 mcg/mL
Loading dose	22 mg/kg (ABW or DW if obese)	24 mg/kg (ABW or DW if obese)
Maintenance dose	13 mg/kg (ABW or DW if obese)	13 mg/kg (ABW or DW if obese)

**Table 5:** 

CrCl (mL/min)	Dosing interval (hours)
Target trough 10–15 mcg/mL	
>100	8
71–100	12
46–70	18
31–45	24
21–30	36
15–20	48
11–14	72
≤10	PRN

Target trough 15–20 mcg/mL	
>80	8
56–80	12
36–55	18
26–35	24
15–25	36
11–14	48
≤10	PRN
