# Supplementary material for: Prospective Trial of a Novel Nomogram to Achieve Updated Vancomycin Trough Concentrations
Source: Interdiscip Perspect Infect Dis. 2013 Sep 12;2013:839456. doi: 10.1155/2013/839456 (PMC3791841; doi:10.1155/2013/839456)
Supplement: Supplementary file 1 — Appendix 1 contains examples of forms utilized during the study. Separate forms were provided for the nomogram and traditionally dosed groups. Also, separate forms were provided for obese and non-obese patients within the nomogram group. [file 839456.f1.pdf]

Appendix I – Study Forms

Non-obese Vancomycin Nomogram Monitoring Form

Place Patient Label:

Age:	
Height:	
Sex:	
Patient Study Number:<br>*Find on randomization sheet	

!

!

!

!

	Column 1	Column 2
**1**	Enter Indication →	
**2**	Enter target trough range (10-15 OR 15-20) →	
**3**	Enter ABW (kg) from randomization sheet<br>***Use for dosing calculations*** →	
**4**	Enter IBW (kg) from randomization sheet →	

Target Trough 10-15 mg/L

**5**	Loading dose (check one):<br><br>Already given = record dose in Box A below<br>OR<br>Calculate loading dose and record in Box A below<br>LD = 22mg/kg x (ABW)
**6**	Maintenance dose:<br>Calculate and enter in Box B<br>MD = 13mg/kg x (ABW)
**7**	Enter initial serum creatinine (mg/dL) in Box C
**8**	Calculate creatinine clearance (mL/min) and enter in Box D<br><br>CrCl = (140 - age)(IBW)* (0.85 in females) / 72 x Scr<br>* use ABW if the patient weighs less than calculated IBW
**9**	Select appropriate dosing interval based on calculated creatinine clearance and enter in Box E

CrCl (mL/min)	Dosing Interval (hrs)
>100	8
71-100	12
46-70	18
31-45	24
21-30	36
15-20	48
11-14	72
! 10	prn

Target Trough 15-20 mg/L

**5**	Loading dose (check one):<br><br>Already given = record dose in Box A below<br>OR<br>Calculate loading dose and record in Box A below<br>LD = 24mg/kg x (ABW)
**6**	Maintenance dose:<br>Calculate and enter in Box B<br>MD = 13mg/kg x (ABW)
**7**	Enter initial serum creatinine (mg/dL) in Box C
**8**	Calculate creatinine clearance (mL/min) and enter in Box D<br><br>CrCl = (140 - age)(IBW)* (0.85 in females) / 72 x Scr<br>* use ABW if the patient weighs less than calculated IBW
**9**	Select appropriate dosing interval based on calculated creatinine clearance and enter in Box E

CrCl (mL/min)	Dosing Interval (hrs)
>80	8
56-80	12
36-55	18
26-35	24
15-25	36
11-14	48
! 10	prn

!

**A**	**Loading Dose**	
**B**	**Maintenance Dose**	
**C**	**Initial Scr**	
**D**	**Creatinine Clearance**	
**E**	**Dosing Interval**	

Obese Vancomycin Nomogram Monitoring Form

Place Patient Label:

Age:	
Height:	
Sex:	
Patient Study Number:<br>*Find on randomization sheet	

!
!
!

	Column 1	Column 2
**1**	Enter Indication →	
**2**	Enter target trough range (10-15 OR 15-20) →	
**3**	Enter DW (kg) from randomization sheet<br>***Use for calculations*** →	

Target Trough 10-15 mg/L

**4**	Loading dose (check one):<br>Already given = record dose in Box A below<br>OR<br>Calculate loading dose and record in Box A below<br>LD = 22mg/kg x (DW)
**5**	Maintenance dose:<br>Calculate and enter in Box B<br>MD = 13mg/kg x (DW)
**6**	Enter initial serum creatinine (mg/dL) in Box C
**7**	Calculate creatinine clearance (mL/min) and enter in Box D<br>CrCl = (140 - age)(DW)* (0.85 in females) / 72 x Scr
**8**	Select appropriate dosing interval based on calculated creatinine clearance and enter in Box E

CrCl (mL/min)	Dosing Interval (hrs)
>100	8
71-100	12
46-70	18
31-45	24
21-30	36
15-20	48
11-14	72
! 10	prn

Target Trough 15-20 mg/L

**4**	Loading dose (check one):<br>Already given = record dose in Box A below<br>OR<br>Calculate loading dose and record in Box A below<br>LD = 24mg/kg x (DW)
**5**	Maintenance dose:<br>Calculate and enter in Box B<br>MD = 13mg/kg x (DW)
**6**	Enter initial serum creatinine (mg/dL) in Box C
**7**	Calculate creatinine clearance (mL/min) and enter in Box D<br>CrCl = (140 - age)(DW)* (0.85 in females) / 72 x Scr
**8**	Select appropriate dosing interval based on calculated creatinine clearance and enter in Box E

CrCl (mL/min)	Dosing Interval (hrs)
>80	8
56-80	12
36-55	18
26-35	24
15-25	36
11-14	48
! 10	prn

!

**A**	**Loading Dose**	
**B**	**Maintenance Dose**	
**C**	**Initial Scr**	
**D**	**Creatinine Clearance**	
**E**	**Dosing Interval**	

Traditional Dosing Vancomycin Monitoring Form

Place Patient Label:

Age:	
Height:	
Sex:	
Patient Study Number:<br>*Find on randomization sheet	

Please check one: Kinetic Dosing (Fill out form below) Global Rph (Print and attach Global Rph Form) Other

	Column 1	Column 2
**1**	Enter ABW (kg) from randomization sheet →	
**2**	Enter IBW (kg) from randomization sheet →	
**3**	Enter DW (kg) if calculated on randomization sheet →	
**3**	Enter Indication →	
**4**	Enter target trough range (10-15 OR 15-20) →	
**5**	Enter initial Scr (mg/dL) in column 2 →	
**6**	Calculate creatinine clearance (mL/min) and enter in column 2 →<br>$CrCl = \frac{(140 - age)(IBW)^*}{72 \times Scr}$ (0.85 in females)<br>* use ABW if the patient weighs less than calculated IBW and DW if obese	
**7**	Calculate and enter k_e<br>$ke = 0.00083(CrCl) + 0.0044$ →	
**8**	Calculate and enter half life →<br>$T_{1/2} = 0.693/k_e$	
**9**	Calculate and enter Vd →<br>Vd = 0.7L/kg x (ABW) or (DW) if obese	
**10**	Loading dose (check one):<br>Already given = record dose →<br>OR<br>Calculate loading dose and record →<br>LD = Vd x desired peak	
**11**	Calculate Dosing Interval and record →<br>$t = \frac{ln\left(\frac{Cpk}{Ctr}\right)}{k}$	
**12**	Calculate Maintenance dose (MD) and record →<br>$MD = Cpeak \times Vd \times (1-e^{-kt})$	
**13**	Calculate estimated peak and record<br>$Cpk\ actual = Cpk\ desired \times \frac{actual\ dose}{theoretical\ dose}$ →	
**14**	Calculate estimated trough and record<br>$Ctrough = Cpk\ actual \times e^{-kt}$ →	
